# From DMEK to Corneal Endothelial Cell Therapy: Technical and Biological Aspects

**DOI:** 10.1155/2018/6482095

**Published:** 2018-08-01

**Authors:** Raffaele Nuzzi, Paola Marolo, Federico Tridico

**Affiliations:** ^1^S.C.U. Ophthalmology Unit, “City of Health and Science” University Hospital, Via Cherasco 23, 10126 Turin, Italy; ^2^Department of Surgical Sciences, University of Turin, Corso Dogliotti 14, 10126 Turin, Italy

## Abstract

The main treatment available for restoration of the corneal endothelium is keratoplasty and DMEK provides faster visual recovery and better postoperative visual acuity when compared to DSAEK. However, the technical challenges related to this technique and the steep technical learning curve seem to prevent the overcoming of DSAEK in favor of DMEK. Furthermore, the outcome of lamellar keratoplasty techniques is influenced by problems related to corneal grafting tissue availability, management, and quality. On the other hand, improvements in the field of cell engineering have opened the way for the use of stem cells-derived corneal endothelial cells with regenerative intent. In this overview, latest findings in endothelial cell engineering are reported, and perspectives of clinical application of mesenchymal stem cells for corneal endothelial replacement and regeneration are evaluated.

## 1. Introduction

Currently, keratoplasty is the main solution for the treatment of diseases involving corneal endothelium. Frequent indications to endothelial corneal grafting include Fuchs' endothelial dystrophy, bullous keratopathy following phacoemulsification, and endothelial disfunction after corneal transplant. Descemet's membrane endothelial keratoplasty (DMEK), introduced in 2006 [1], represents the most novel technique for endothelial keratoplasty. It differs from Descemet's stripping-automated endothelial keratoplasty (DSAEK) for the use of a grafted material that includes no corneal stroma but only endothelium and Descemet membrane. The graft can thus be introduced into the anterior chamber and applied to the posterior stroma through the injection of an air bubble. The graft rejection risk is lower in DMEK when compared to DSAEK, and several studies have demonstrated that DMEK provides faster visual recovery and better postoperative visual acuity than DSAEK [2–5]. Furthermore, as of today, the rate of primary graft failure after DMEK seems lower if compared to DSEK [6–8].

Despite the promising outcomes of this new technique, DMEK is affected by several technical difficulties. In first instance, the surgical complexity (e.g., because of the thinner tissue used, graft unfolding can be more challenging) and its steep learning curve discourage many surgeons from leaving DSAEK in favor of this technique [9–11]. In addition, a higher graft detachment rate after DMEK might lead to more frequent rebubbling or graft repositioning [12]. Another problem is primary endothelial cell loss, which seems to be related to surgeon experience [13]. Loss of endothelial cells is higher in the early postoperative time after DMEK and around 7% per year in the following period [14].

Lately, several improvements have been made in the realization of techniques to isolate and administer human corneal cells as an alternative to keratoplasty [15]. Emerging strategies of tissue engineering for corneal endothelial applications focus on transplantable endothelial cells production [16].

Nowadays, cell therapy is focused on culture of corneal endothelial cells retrieved from donors, followed by grafting in the donor's cornea. The current research is focusing on the expansion of human corneal endothelial cells to overcome the shortage of donor tissues [15]; however, bioengineered corneal endothelium could lead to promising perspectives for potential applications with regenerative intent [17].

## 2. Current Findings in Cell Application for Corneal Endothelial Deficiency

Several studies have been conducted investigating the in vitro expansion of corneal endothelial cells (CECs) derived from humans [18, 19] and animal models [20–24]. Human CECs can be isolated from donor corneas with the application of EDTA, trypsin, or collagenase II. Furthermore, through testing of different culturing factors (such as basal culture media, additives [25, 26], and methods of media modulation [25, 27]), several growth environments have been developed in order to allow the expansion of human CECs (such as human corneal stroma, collagen, amniotic membrane, and biodegradable polymers) [28].

As of today, different applicative approaches have been proposed for human CECs: monolayered cell sheets, cellular injection therapy, and cell-carrying systems [25]. Localization of cultivated human CECs (delivered via intracameral injection) onto the posterior corneal surface has been tested through ferromagnetic induction [29, 30] and gravity due to prone posture [18], or a combination of these methods. Previous studies from Mimura et al. evaluated the treatment of corneal endothelial deficiency in rabbit models with intracameral injection of sphere colonies of corneal endothelial progenitor cells [30]. On the other hand, ultrathin sheets of human corneal endothelial cells have been transplanted with DSAEK devices in animal models. Recently, the function and clinical adaptability of isolated primary human corneal endothelial cells have been evaluated in a preclinical rabbit model of endothelial keratopathy, via a tissue-engineered endothelial keratoplasty approach, with positive outcomes regarding corneal thickness reduction [31]. However, this method may prove to be too challenging to be clinically applicable, since an excessively thin sheet may be difficult to handle. For this reason, cells injections seem more technically feasible. Also, several cell-seeded scaffolds have been evaluated for corneal transplantation (composed of different materials such as porcine Descemet's membrane [32, 33], chitosan [34], hydrogel lens [35], and paramagnetic microspheres [36]) promoting transferring of cultivated cells into target corneas. In accordance with these considerations, a clinical trial was started in 2013 to investigate the application of cultured endothelial cell injections, supplemented with a ROCK inhibitor (which showed to be useful for endothelial wound healing [37]) with improvements in endothelial cell density, corneal thickness reduction, and visual acuity (UMIN000012534) [38, 39].

## 3. Stem Cells for Corneal Endothelium Diseases

Although derivation of human corneal endothelial cells from embryonic stem cells (with their broad differentiation potential) has been reported [40], several concerns surrounding the use of this type of stem cells, on the ethical level—regarding retrieval/donation of oocytes and extraction from the destruction of embryos—and also in terms of safety with the high risk of teratoma development, seem to limit its clinical application [41]. However, a transcriptome analysis on embryonic-derived endothelial stem cells revealed the expression of several markers shared with CECs (such as ZO1, Col8a, and CRY1), with promising perspectives of future applications of stem cells-derived CECs [42]. Given these considerations, an impressive effort has been dedicated to the identification of alternative sources, more suitable for corneal endothelial cells production.

It has been shown that induced pluripotent stem cells (iPSCs) extracted from monkeys can be modified into corneal endothelial cells (through the use of an endothelium-deriving medium including GSK-3-beta inhibitor, retinoic acid, and a ROCK inhibitor) capable of regulating corneal stromal transparency after transplantation into rabbit eyes [43]. However, relevant matters that currently hinder the use of iPSCs are the extremely low rate at which adult somatic cells can be altered in order to obtain iPSCs and the specific conditions allowing the differentiation of corneal endothelial cells from human iPSCs, which have not been fully discovered yet. Taking into consideration also the potential oncogenic risk related to iPSCs [44], an important work is still required to perfect iPSCs differentiation before their safe and efficient application for corneal tissue engineering can be achieved.

Mesenchymal stem cells (MSCs) can be easily obtained from different human tissues, and their application (similarly to other pluripotent cell types) seems more feasible if compared to primary human corneal endothelial cells, which have limited proliferative capacities [45]. As of today, MSCs have been administered through two different delivery routes: local (through direct injection or cell-seeded scaffolds) and systemic (intravenous or intra-arterial introduction) [46]. As multipotent MSCs and iPSCs are derived from adult tissues, they can be used without the ethical problems surrounding embryonic stem cells. In addition, autologous MSCs avoid the need for immune-suppressive drugs to prevent rejection of allogenic grafts.

The potential of phenotypical alteration of mesenchymal stem cells towards human corneal endothelial-like cells is based on the fact that during eye development in many species, including humans, corneal endothelial cells differentiate from neural crest-derived periocular mesenchymal cells (this embryological development is supported also by several immunohistochemical findings) [47–49]. Moreover, MSCs and human CECs share some mesenchymal features (since CECs are able to alter their shape into a fibroblast-like one and can produce type IV collagen, in presence of FGF and fibroblastic extracellular matrix [50, 51]) and are able to express adhesion proteins such ZO1 and N-cadherin [52], although they are considered as different cell types. However, as of today, directed differentiation of CECs has not been clearly identified and the ability to clearly detect a definitive CEC clone derived from pluripotent or stem cells sources is still insufficient.

## 4. Potential Applications of MSCs for Endothelial Replacement

MSCs open many perspectives for cell-based clinical applications, due to their regenerating ability and their potential to differentiate into many different cell types [53, 54]. MSCs can be easily retrieved from different sources like bone marrow (BM-MSC), adipose tissue (AT-MSC), skeletal muscle, dental pulp, umbilical cord (U-MSCs), and blood from umbilical cord (UCB-MSCs) [55–62]. However, the ideal source tissue for endothelial cell differentiation remains to be discovered. In fact, MSCs derived from different sources show similar morphology but different colony generation rate, proliferation, and differentiation capacities. For example, UCB-MSCs possess the highest differentiation capacity, but the lowest colony generation frequency, while AT-MSCs have the highest colony generation frequency and the lowest proliferation capacity goes to BM-MSCs [63]. Moreover, only a limited number of MSCs can be extracted from adult tissues (generally, a 5 ml bone marrow aspirate may contain between 2,500 and 6,000 MSCs) [64]. Even if this amount may be sufficient for repairing corneal endothelial lesions, avoiding excessive manipulation of the cell product, there is not an established number of cells to initiate the treatment, and the expansion of MSCs can still be required for clinical application. However, classical monolayer expansion techniques do not preserve MSCs progenitor potency, but novel alternative methods (such as 3D dynamic cultures, scaffolds, and growth factors applications and hypoxia modulation) can enhance the efficacy of expanded cells for clinical application [65].

**Figure 1:**
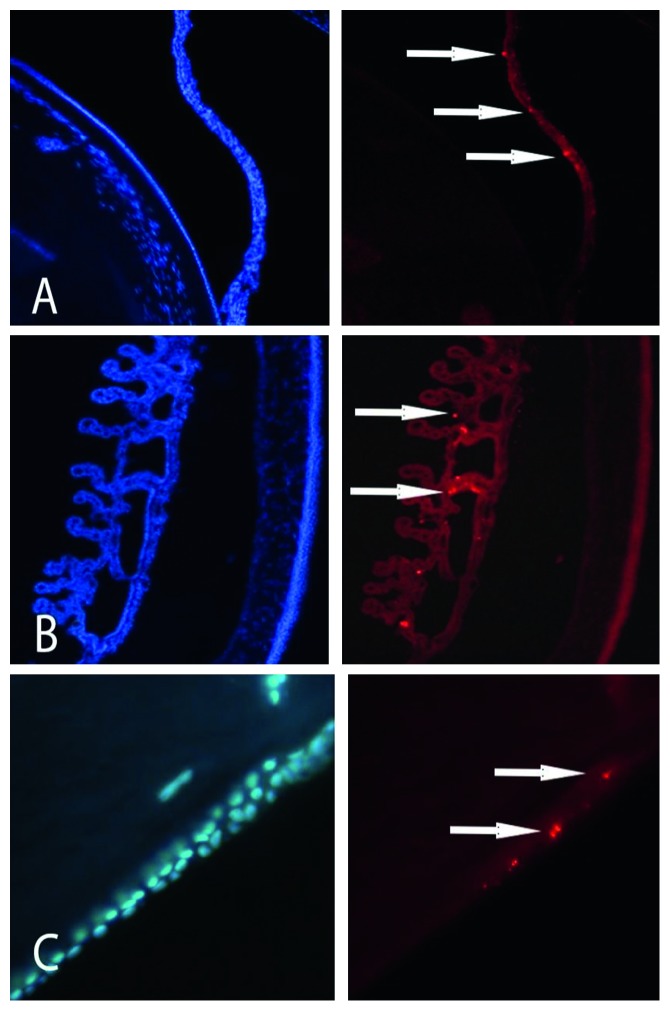
Mesenchymal stem cells introduced in the anterior chamber of murine models can migrate towards the (A) iris, (B) ciliary body, and (C) lens. Migrated cells (indicated by the arrows in the images on the right) could be observed in the anterior segment for 6 weeks. Images on the right are highlighted with a filter specific for bisbenzimide, while images on the right are visualized with a filter specific for Dil Stain.

It has been demonstrated that human BM-MSCs can differentiate into epithelial corneal cells in vivo and into corneal keratocytes in vitro and AT-MSCs can differentiate into epithelial corneal cells in vitro, while human U-MSCs can differentiate into corneal keratocytes in vivo [66–68]. An appealing aspect of MSCs is their ability to aim mainly to injured areas, where they can differentiate into different cell types in accordance to the surrounding microenvironment [54]. We had also performed preliminary evaluations, highlighting the ability of MSCs to survive, migrate, and integrate at the level of cornea, iris, ciliary body, and lens in eyes of healthy mice after injection in the anterior chamber [69] (Figure 1). Moreover, the application of mesenchymal stem cells, with their high plasticity potential and relative safety, avoids the ethical and biological concerns related to the use of embryonic stem cells and iPSCs [70, 71].

The optimal MSCs delivery strategy for clinical application in corneal diseases is represented by local administration through different methods, since it is also the main technique currently used for hCECs. Several studies have focused on the application of mesenchymal stem cells for corneal diseases treatment. For example, UCB-MSCs have been transplanted into corneas of mice leading to improved corneal transparency and increased stromal thickness [68], and autologous bone marrow MSCs have been used to replace corneal endothelium of rabbits in vivo [72]. These studies provided encouraging results, even if the phenotype of implanted cells was examined using morphological techniques (live confocal imaging and scanning electron microscopy). Joyce et al. reported that the phenotype of UCB-MSCs could be altered toward endothelial cell-like cells and that these modified cells tend to “home” to injured areas of ex-vivo corneal endothelium disease models. Moreover, grafting of these modified MSCs did not occur onto normal areas of corneal endothelium or Descemet's membrane, suggesting a high-specificity action of these cells [73]. Homing of MSCs to wounded endothelium is probably due to chemotaxis induced by tumor necrosis factor-*α* (TNF-*α*) and expression of adhesion molecules on the surface of endothelial cells (such as ICAM-1 and ELAM-1), both occurring during inflammation [74, 75] and promoting migration and adhesion of MSCs in injured areas [75, 76]. Also, Zo1 and N-cadherin expressions, used to examine the presence of MSCs, were more distributed at the level of the damaged area [73]. However the presence, even if in a smaller quantity, of the same proteins in untreated cultures raises some doubts regarding the differentiation process of MSCs into endothelial cells. Moreover, the typical hexagonal shape of corneal endothelial cells was not observed in the endothelial-like cells derived from MSCs and the molecular basis for the complete transition from mesenchymal to endothelial type is still not known. Even if further research is needed to better understand the environmental conditions leading to an adequate differentiation, MSC can be considered as a potential candidate for corneal endothelial cells replacement.

**Figure 2:**
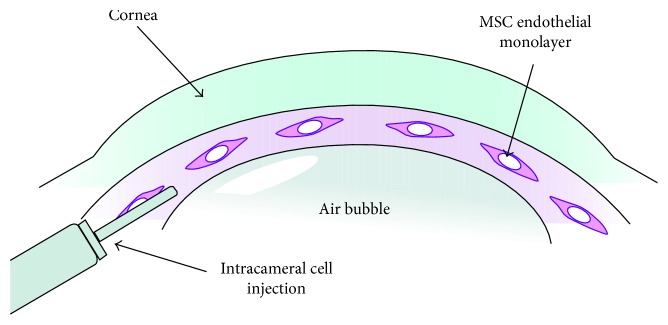
Schematic diagram showing injection of mesenchymal stem cells in the anterior chamber with a cornea-air interface (currently under evaluation in animal models).

MSCs or endothelial-like cells derived from MSCs can be applied with techniques similar to those adopted previously for other cell types, avoiding the limits of human corneal endothelial cells, which can't be expanded indefinitely. Moreover, MSCs can also be cultivated to create an ultrathin sheet for endothelial replacement [77], but with implicit technical difficulties for the surgeon (given the difficult management). In the light of all these findings, we are currently evaluating the application of MSCs through intracameral injection in murine models with two modalities (Figure 2): the first one features total asportation of the diseased endothelium (with Descemet's membrane sparing), followed by the injection of a small quantity of mesenchymal stem cells in the anterior chamber while the other one features the injection of MSCs without previous endothelial asportation, with regenerative and repairing intent, especially at the level of the areas where cell elements are absent.

Both modalities should be carried out with a paralimbal injection, through a dedicated “ad hoc” syringe, on central and/or peripheral corneal areas, after the realization in the anterior chamber of an air-stroma (for the first method) or air-endothelium (for the latter) interface. It will be possible to inject a total amount of 1000/2000/5000 MSCs (dispensed in multiple administrations, three months away from each other, in relation to cell attachment, migration, and survival and depending on a case-by-case evaluation). Integration of MSCs is related to the quantity of injected cells with the possibility of repeated injections.

## 5. Discussion and Applicative Aspects

Availability of donors for endothelial keratoplasty is an emerging problem that must be addressed. Corneal graft tissue derived from cadavers must meet stringent adequacy criteria that include serological tests (in selected cases) and medical history of the donor [78–80]. However, many potential donor corneas, often from elderly donors, are rejected for transplantation because of their lower endothelial cell count and possible age-related alterations. Moreover, availability of corneas can be also affected by cultural, logistical, and technical difficulties [81], and long postmortem time or damage occurred during handling of donor corneas can possibly lead to unsuitable graft material [82]. These are some of the impelling problems that must be taken into account in conjunction with global shortage of suitable corneal tissues. To further alleviate the growing demand for grafting quality tissue, a considerable clinical interest is being amplified in the development of tissue engineering as a suitable alternative for corneal graft [81].

The application of mesenchymal stem cells for corneal endothelial replacement can be considered as a promising perspective, even if its potential still remains to be functionally assessed with additional studies on in vivo animal models. First of all, it reduces the need for donor corneas, saving resources which are more and more limited in time. Moreover, autologous mesenchymal stem cells can be extracted from the same patient, which can act both as donor and receiving subject, further lowering rejection rates and immunological reactions. Since these cells can be injected in the anterior chamber, technical issues related to surgery are reduced (with no corneal flap that needs to be carefully managed). Furthermore, repeated injections can be performed, if needed (such in case of failure or recurrence), after a second cell extraction or after MSCs expansion (that can function as a reservoir sample). MSCs adhesion can also be promoted by coating with antibodies specific for endothelial adhesion proteins, improving targeting of MSCs to wounded areas [76]. In addition, MSC injections can prevent endothelial transplant failure due to cell deficiency which can occur in donor corneas for age-related modifications. For this reason, endothelial cell therapy can be considered also as an adjuvant therapy after DMEK and not only as a first-step approach. Autologous MSCs engraftment can be evaluated in clinical trials on humans with the additional advantage given by the possibility of patients prone positioning, which should facilitate their integration (always preceded by a case-by-case setting). Another problem following DMEK and DSAEK is represented by optical aberrations, due to stromal/donor flap interface. Since the interface can be significantly reduced with the application of mesenchymal stem cells, optical aberration and hyperopic shift should be avoided.

If a translational approach was promoted, and corneal endothelial cell engineering was adopted in a widespread fashion, social and economic costs would be progressively lowered, with saving of donor corneas, surgical material, and management expenses. Currently, specific intracellular signals leading to morphologic changes of MSCs are not known; hence, further investigation of the molecular basis related to mesenchymal-to-epithelial transition of MSCs is needed. Other issues that remain to be addressed before clinical application of mesenchymal stem cells for corneal endothelium replacement are the definition of the best source to obtain MSCs and best conditions for cell expansion, suppression of differentiation to undesired cell types, and homing to damaged corneal areas. Additional studies, including in vivo testing, are now needed to identify the specific conditions that would best support the ability of MSCs to replace corneal endothelial cells lost due to damage or disease as means of restoring corneal transparency. Finally, adverse events related to MSCs application are rare and often related to the microenvironment in which MSCs engraft—leading to unwanted differentiation of transplanted cells—or the presence of preexisting tumors whose growth may be promoted by immunosuppression provided by MSCs [83]. Even if autologous MSCs can be considered safe in terms of immunogenicity and de-novo malignancy development [84], long-term clinical trials are still required to evaluate differentiation concerns and clinical efficacy in human corneas.

## References

[1] Melles G. R., Ong T. S., Verves B., van der Wees J. (2006). Descemet membrane endothelial keratoplasty (DMEK). *Cornea*.

[2] Tourtas T., Laaser K., Bachmann B. O. (2012). Descemet membrane endothelial keratoplasty versus Descemet stripping automated endothelial keratoplasty. *American Journal of Ophthalmology*.

[3] Bhandari V., Reddy J. K., Relekar K., Prabhu V. (2015). Descemet’s stripping automated endothelial keratoplasty versus Descemet’s membrane endothelial keratoplasty in the fellow eye for fuchs endothelial dystrophy: a retrospective study. *BioMed Research International*.

[4] Guerra F. P., Anshu A., Price M. O., Price F. W. (2011). Endothelial keratoplasty: fellow eyes comparison of Descemet stripping automated endothelial keratoplasty and Descemet membrane endothelial keratoplasty. *Cornea*.

[5] Hori J., Joyce N. C., Streilein J. W. (2000). Immune privilege and immunogenicity reside among different layers of the mouse cornea. *Investigative Ophthalmology and Visual Science*.

[6] Deng S. X., Lee W. B., Hammersmith K. M. (2018). Descemet membrane endothelial keratoplasty: safety and outcomes: a report by the American Academy of Ophthalmology. *Ophthalmology*.

[7] Lee W. B., Jacobs D. S., Musch D. C. (2009). Descemet’s stripping endothelial keratoplasty: safety and outcomes: a report by the American Academy of Ophthalmology. *Ophthalmology*.

[8] Maier P., Reinhard T., Cursiefen C. (2013). Descemet stripping endothelial keratoplasty—rapid recovery of visual acuity. *Deutsches Aerzteblatt Online*.

[9] Terry M. A. (2012). Endothelial keratoplasty: why aren’t we all doing Descemet membrane endothelial keratoplasty?. *Cornea*.

[10] Price M. O., Price F. W. (2010). Descemet membrane endothelial keratoplasty. *International Ophthalmology Clinics*.

[11] Price F. W., Price M. O. (2009). *DSEK: What You Need to Know about Endothelial Keratoplasty*.

[12] Dapena I., Ham L., Melles G. R. (2009). Endothelial keratoplasty: DSEK/DSAEK or DMEK-the thinner the better?. *Current Opinion in Ophthalmology*.

[13] Koenig S. B., Covert D. J., Dupps W. J., Meisler D. M. (2007). Visual acuity, refractive error, and endothelial cell density six months after Descemet stripping and automated endothelial keratoplasty (DSAEK). *Cornea*.

[14] Baydoun L., Tong C. M., Tse W. W. (2012). Endothelial cell density after Descemet membrane endothelial keratoplasty: 1 to 5-year follow-up. *American Journal of Ophthalmology*.

[15] Navaratnam J., Utheim T. P., Rajasekhar V. K., Shahdadfar A. (2015). Substrates for expansion of corneal endothelial cells towards bioengineering of human corneal endothelium. *Journal of Functional Biomaterials*.

[16] Yuan S., Fan G. (2015). Stem cell-based therapy of corneal epithelial and endothelial diseases. *Regenerative Medicine*.

[17] Zavala J., López Jaime G. R., Rodríguez Barrientos C. A., Valdez-Garcia J. (2013). Corneal endothelium: developmental strategies for regeneration. *Eye*.

[18] Peh G. SL., Toh K. P., Wu F. Y. (2011). Cultivation of human corneal endothelial cells isolated from paired donor corneas. *PLoS One*.

[19] Peh G. SL., Chng Z., Ang H. P. (2015). Propagation of human corneal endothelial cells: a novel dual media approach. *Cell Transplantation*.

[20] Koizumi N., Sakamoto Y., Okumura N. (2007). Cultivated corneal endothelial cell sheet transplantation in a primate model. *Investigative Opthalmology and Visual Science*.

[21] Okumura N., Koizumi N., Ueno M. (2011). The new therapeutic concept of using a rho kinase inhibitor for the treatment of corneal endothelial dysfunction. *Cornea*.

[22] Okumura N., Koizumi N., Ueno M. (2011). Enhancement of corneal endothelium wound healing by Rho-associated kinase (ROCK) inhibitor eye drops. *British Journal of Ophthalmology*.

[23] Okumura N., Koizumi N., Ueno M. (2012). ROCK inhibitor converts corneal endothelial cells into a phenotype capable of regenerating in vivo endothelial tissue. *American Journal of Pathology*.

[24] Li S., Wang C., Dai Y. (2013). The stimulatory effect of ROCK inhibitor on bovine corneal endothelial cells. *Tissue and Cell*.

[25] Soh Y. Q., Peh G. S. L., Mehta J. S. (2017). Translational issues for human corneal endothelial tissue engineering. *Journal of Tissue Engineering and Regenerative Medicine*.

[26] Vianna L. M. M., Kallay L., Toyono T. (2015). Use of human serum for human corneal endothelial cell culture. *British Journal of Ophthalmology*.

[27] Sha X., Liu Z., Song L. (2013). Human amniotic epithelial cell niche enhances the functional properties of human corneal endothelial cells via inhibiting P53–survivin–mitochondria axis. *Experimental Eye Research*.

[28] Choi J. S., Kim E. Y., Kim M. J. (2014). Factors affecting successful isolation of human corneal endothelial cells for clinical use. *Cell Transplantation*.

[29] Mimura T., Shimomura N., Usui T. (2003). Magnetic attraction of iron-endocytosed corneal endothelial cells to Descemet’s membrane. *Experimental Eye Research*.

[30] Mimura T., Yamagami S., Usui T. (2005). Long-term outcome of iron-endocytosing cultured corneal endothelial cell transplantation with magnetic attraction. *Experimental Eye Research*.

[31] Peh G. SL., Ang H.-P., Lwin C. N. (2017). Regulatory compliant tissue-engineered human corneal endothelial grafts restore corneal function of rabbits with bullous keratopathy. *Scientific Reports*.

[32] Shao C., Fu Y., Lu W., Fan X. (2011). Bone marrow-derived endothelial progenitor cells: a promising therapeutic alternative for corneal endothelial dysfunction. *Cells Tissues Organs*.

[33] Schwartzkopff J., Bredow L., Mahlenbrey S., Boehringer D., Reinhard T. (2010). Regeneration of corneal endothelium following complete endothelial cell loss in rat keratoplasty. *Molecular Vision*.

[34] Wang T.-J., Wang I.-J., Lu J.-N., Young T.-H. (2012). Novel chitosan-polycaprolactone blends as potential scaffold and carrier for corneal endothelial transplantation. *Molecular Vision*.

[35] Mohay J., Wood T. O., McLaughlin B. J. (1997). Long-term evaluation of corneal endothelial cell transplantation. *Transactions of the American Ophthalmological Society*.

[36] Patel S. V., Bachman L. A., Hann C. R., Bahler C. K., Fautsch M. P. (2009). Human corneal endothelial cell transplantation in a human ex vivo model. *Investigative Opthalmology and Visual Science*.

[37] Okumura N., Koizumi N., Kay E. P. (2013). The ROCK inhibitor eye drop accelerates corneal endothelium wound healing. *Investigative Opthalmology and Visual Science*.

[38] Okumura N., Kinoshita S., Koizumi N. (2014). Cell-based approach for treatment of corneal endothelial dysfunction. *Cornea*.

[39] Kinoshita S., Koizumi N., Ueno M. (2018). Injection of cultured cells with a ROCK inhibitor for bullous keratopathy. *New England Journal of Medicine*.

[40] Pang K., Zhang K., Zhu J. (2015). Differentiation of human embryonic stem cells to corneal epithelium and endothelium like cells for cornea replacement construction. *Investigative Opthalmology and Visual Science*.

[41] Wu J., Izpisua Belmonte J. C (2015). Dynamic pluripotent stem cell states and their applications. *Cell Stem Cell*.

[42] Song Q., Yuan S., An Q. (2016). Directed differentiation of human embryonic stem cells to corneal endothelial cell-like cells: a transcriptomic analysis. *Experimental Eye Research*.

[43] Hatou S., Yoshida S., Higa K. (2013). Corneal endothelial cells derived from monkey iPS cells: a short-term evaluation. *Investigative Opthalmology and Visual Science*.

[44] Gutierrez-Aranda I., Ramos-Mejia V., Bueno C. (2010). Human induced pluripotent stem cells develop teratoma more efficiently and faster than human embryonic stem cells regardless of the site of injection. *Stem Cells*.

[45] Joyce N. C. (2012). Proliferative capacity of corneal endothelial cells. *Experimental Eye Research*.

[46] Kean T. J., Lin P., Caplan A. I., Dennis J. E. (2013). MSCs: delivery routes and engraftment, cell-targeting strategies, and, immune modulation. *Stem Cells International*.

[47] Cvekl A., Tamm E. R. (2004). Anterior eye development and ocular mesenchyme: new insights from mouse models and human diseases. *Bioessays*.

[48] Hayashi K., Sueishi K., Tanaka K., Inomata H. (1986). Immunohistochemical evidence of the origin of human corneal endothelial cells and keratocytes. *Graefe’s Archive for Clinical and Experimental Ophthalmology*.

[49] Böhnke M., Vogelberg K., Engelmann K. (1998). Detection of neuronespecific enolase in long-term cultures of human corneal endothelium. *Graefe’s Archive for Clinical and Experimental Ophthalmology*.

[50] Neufeld A. H., Jumblatt M. M., Matkin E. D., Raymond G. M. (1986). Maintenance of corneal endothelial cell shape by prostaglandin E2: effects of EGF and indomethacin. *Investigative Opthalmology and Visual Science*.

[51] Hsieh P., Baum J. (1985). Effects of fibroblastic and endothelial extracellular matrices on corneal endothelial cells. *Investigative Opthalmology and Visual Science*.

[52] Petroll W. M., Jester J. V., Bean J., Cavanagh H. D. (1999). The spatial organization of apical junctional complex-associated proteins in feline and human corneal endothelium. *Current Eye Research*.

[53] Baksh D., Song L., Tuan R. S. (2004). Adult mesenchymal stem cells: characterization, differentiation, and application in cell and gene therapy. *Journal of Cellular and Molecular Medicine*.

[54] Chamberlain G., Fox J., Ashton B., Middleton J. (2007). Concise review: mesenchymal stem cells: their phenotype, differentiation capacity, immunological features, and potential for homing. *Stem Cells*.

[55] Pittenger M. F., Mackay A. M., Beck S. C. (1999). Multilineage potential of adult human mesenchymal stem cells. *Science*.

[56] Zuk P. A., Zhu M., Ashjian P. (2002). Human adipose tissue is a source of multipotent stem cells. *Molecular Biology of the Cell*.

[57] De Bari C., Dell’Accio F., Tylzanowski P., Luyten F. P. (2001). Multipotent mesenchymal stem cells from adult human synovial membrane. *Arthritis and Rheumatism*.

[58] Uezumi A., Ojima K., Fukada S. (2006). Functional heterogeneity of side population cells in skeletal muscle. *Biochemical and Biophysical Research Communications*.

[59] Laino G., Graziano A., d’Aquino R. (2006). An approachable human adult stem cell source for hard-tissue engineering. *Journal of Cellular Physiology*.

[60] Wang H. S., Hung S. C., Peng S. T. (2004). Mesenchymal stem cells in the Wharton’s jelly of the human umbilical cord. *Stem Cells*.

[61] Romanov Y. A., Svintsitskaya V. A., Smirnov V. N. (2003). Searching for alternative sources of postnatal human mesenchymal stem cells: candidate MSC-like cells from umbilical cord. *Stem Cells*.

[62] Erices A., Conget P., Minguell J. J. (2000). Mesenchymal progenitor cells in human umbilical cord blood. *British Journal of Haematology*.

[63] Chang Y. J., Shih D. T., Tseng C. P., Hsieh T. B., Lee D. C., Hwang S. M. (2006). Disparate mesenchyme-lineage tendencies in mesenchymal stem cells from human bone marrow and umbilical cord blood. *Stem Cells*.

[64] Cuthbert R., Boxall S. A., Tan H. B. (2012). Single platform quality control assay to quantify multipotential stromal cells in bone marrow aspirates prior to bulk manufacture or direct therapeutic use. *Cytotherapy*.

[65] Hoch A. I., Leach J. K. (2014). Concise review: optimizing expansion of bone marrow mesenchymal stem/stromal cells for clinical applications. *Stem Cells Translational Medicine*.

[66] Ma Y., Xu Y., Xiao Z. (2006). Reconstruction of chemically burned rat corneal surface by bone marrow-derived human mesenchymal stem cells. *Stem Cells*.

[67] Nieto-Miguel T., Galindo S., Reinoso R. (2013). In vitro simulation of corneal epithelium microenvironment induces a corneal epithelial-like cell phenotype from human adipose tissue mesenchymal stem cells. *Current Eye Research*.

[68] Liu H., Zhang J., Liu C. Y. (2010). Cell therapy of congenital corneal diseases with umbilical mesenchymal stem cells: lumican null mice. *PLoS One*.

[69] Nuzzi R. Prospettive di terapia cellulare oculare.

[70] Kögler G., Sensken S., Airey J. A. (2004). A new human somatic stem cell from placental cord blood with intrinsic pluripotent differentiation potential. *Journal of Experimental Medicine*.

[71] Markov V., Kusumi K., Tadesse M. G. (2007). Identification of cord blood-derived mesenchymal stem/stromal cell populations with distinct growth kinetics, differentiation potentials, and gene expression profiles. *Stem Cells and Development*.

[72] Liu X. W., Zhao J. L. (2007). Transplantation of autologous bone marrow mesenchymal stem cells for the treatment of corneal endothelium damages in rabbits. *[Zhonghua Yan Ke Za Zhi] Chinese Journal of Ophthalmology*.

[73] Joyce N. C., Harris D. L., Markov V., Zhang Z., Saitta B. (2012). Potential of human umbilical cord blood mesenchymal stem cells to heal damaged corneal endothelium. *Molecular Vision*.

[74] Trinh L., Brignole-Baudouin F., Labbé A., Raphaël M., Bourges J-L, Baudouin C. (2008). The corneal endothelium in an endotoxin-induced uveitis model: correlation between in vivo confocal microscopy and immunohistochemistry. *Molecular Vision*.

[75] Meirelles Lda S., Fontes A. M., Covas D. T., Caplan A. I. (2009). Mechanisms involved in the therapeutic properties of mesenchymal stem cells. *Cytokine and Growth Factor Reviews*.

[76] Ko I. K., Kean T. J., Dennis J. E. (2009). Targeting mesenchymal stem cells to activated endothelial cells. *Biomaterials*.

[77] Yamagami S., Mimura T., Yokoo S., Takato T., Amano S. (2006). Isolation of human corneal endothelial cell precursors and construction of cell sheets by precursors. *Cornea*.

[78] Armitage W. J., Moss S. J., Easty D. L. (1990). Supply of corneal tissue in the United Kingdom. *British Journal of Ophthalmology*.

[79] McColgan K. (2009). Corneal transplant surgery. *Journal of Perioperative Practice*.

[80] Builles N., Kodjikian L., Burillon C. (2006). Major endothelial loss from corneas in organ culture: importance of second endothelial count. *Cornea*.

[81] Ruberti J. W., Zieske J. D. (2008). Prelude to corneal tissue engineering gaining control of collagen organization. *Progress in Retinal and Eye Research*.

[82] Engelmann K., Bednarz J., Valtink M. (2004). Prospects for endothelial transplantation. *Experimental Eye Research*.

[83] Volarevic V., Markovic B. S., Gazdic M. (2018). Ethical and Safety Issues of Stem Cell-Based Therapy. *International Journal of Medical Sciences*.

[84] Lalu M. M., McIntyre L., Pugliese C. (2012). Safety of cell therapy with mesenchymal stromal cells (safecell): a systematic review and meta-analysis of clinical trials. *PLoS One*.

